# Children still exposed to high rates of unhealthy food advertising in Austria: does self-regulation work?

**DOI:** 10.1017/S1368980026102171

**Published:** 2026-02-26

**Authors:** Felizitas Moll, Bettina Meidlinger, Irene Wallisch, Katrin Seper-Nagl, Magdalena Muc, Mimi Tatlow-Golden, Alexandra Wolf

**Affiliations:** 1 Division Integrated Risk Assessment, Data and Statistics, Centre for Nutrition and Disease Prevention, Austrian Agency for Health and Food Safety (AGES), Spargelfeldstraße 191, 1220 Vienna, Austria; 2 Faculty of Wellbeing, Education and Language Studies, The Open University, Stuart Hall Building, Walton Hall, Milton Keynes MK7 6AA, UK

**Keywords:** Self-regulation, High in fat, sugar and/or salt foods, Marketing, Children, TV monitoring

## Abstract

**Objective:**

To assess the exposure of Austrian children to TV high in fat, sugar and/or salt (HFSS) food and beverage ads and identify changes in HFSS food advertising after the implementation of self-regulatory measures of marketing restriction.

**Design:**

All ads shown on five popular TV channels for Austrian children/teenagers were coded over 4 d (360 h) using the WHO TV Monitoring Protocol, to identify food/beverage marketing, marketing strategies, target audience and presence in peak viewing times. Nutrient analysis was performed using nutrient profile models (NPM), which classify foods as permitted or not permitted for marketing to children: WHO EURO NPM for international comparability and Austria’s NPM for local regulatory compliance. Results were compared with pre-regulatory Austrian TV monitoring data.

**Setting:**

Austria.

**Participants:**

None.

**Results:**

Of 9099 ads captured, 17·0 % were for foods and beverages. Most promoted products not permitted for marketing to children according to WHO EURO NPM (81·8 %) and Austria’s NPM (83·8 %). On all channels, the advertising rate for food ads rose throughout the day, culminating during child/teen peak viewing times in the evening. A mix of marketing strategies and persuasive appeals was used; emotional themes (e.g. friendship, holidays and enjoyment) were more common in not permitted ads, compared with permitted ads. Not permitted ads featured elements appealing to children/teenagers significantly more often than permitted ads.

**Conclusions:**

Despite self-regulatory measures of marketing restriction, children and teenagers in Austria are still exposed to a high number of advertisements for HFSS foods using impactful emotional marketing strategies on TV. To protect children from this influence, further regulations are called for.

Childhood obesity increases the risk of developing non-communicable diseases such as heart disease, diabetes or cancer and of experiencing psychological and psychosocial impacts^(1)^. Although some European countries have recently seen numbers stagnating or even declining^(2)^, the global number of children living with obesity keeps rising. Since childhood obesity often persists into adulthood, the overall issue remains alarming, with 43 % of overweight adults worldwide^(3)^. This raises the question of whether effective interventions are yet to be made.

Previous research has indicated that extensive commercial media consumption in children correlates with higher body weight through purchasing and consumption^(4,5)^ and that exposure to unhealthy food advertising influences children’s food intake^(6)^. Aggressive marketing techniques create customer brand preferences, particularly in children and adolescents^(7,8)^. Despite the rise of streaming platforms, traditional TV still plays a significant role in Austria, being watched by 63 % of children and 55 % of teenagers daily^(9,10)^.

In 2010, WHO member states endorsed resolution 63·14 to restrict the marketing and promotion of food products high in fat, sugar and/or salt (HFSS foods) to children worldwide, yet progress has been very slow. The EU Audiovisual Media Services Directive (Directive 2018/1808, AVMSD) determines that children should be protected from ‘*inappropriate audiovisual commercial communications*’ for HFSS foods, ‘*accompanying or included in children’s programmes*’. It recommends ‘*reduc[ing] the exposure of children*’ to such advertisements (art 9, para 4).

The directive encourages self-regulation through codes of conduct, such as the Code of Ethics of the Austrian Advertising Industry^(11)^. This code was implemented by the Austrian Advertising Council in 2021, defining children as ‘*persons before the age of 12*’ (p.20) and claiming that ‘*food advertising plays a subordinate role*’ in the ‘*multi-causal problem of overweight in society*’ (p.24). It is restricted to ads ‘*immediately before, after or during programmes […] aimed at children*’ and ads that, among other factors, ‘*abuse children’s confidence in the quality of the products*’ or ‘*discourage a healthy, active lifestyle*’ (p.22). It is a complaint-based reactive system, which can apply recognised guidelines like nutrient profile models (NPM) to determine the nutritional adequacy of audiovisual food advertisements directed at children in case of a complaint.

In 2021, the Austrian Nutritional Commission issued and recommended the Austrian (AT) NPM^(12)^, based on the WHO-Europe model version of 2015^(13)^, suggesting that before, during and after children’s TV programmes, food and beverages classified as not permitted for marketing to children should not be advertised. However, the present regulations mention only general dietary guidelines to determine the healthfulness of products, not the NPM. Despite clear evidence and guidelines recommending mandatory restrictions^(14,15)^, all regulations, recommendations and NPM are to this date voluntary. In addition, these regulations are accompanied by the EU Pledge Nutrition Criteria^(16)^, a self-regulatory approach by twenty-three leading food and beverage companies, which restricts food and beverage marketing to children under 13 years of age on TV, print, digital and other media.

In 2014, before the Austrian Code of Ethics and NPM were issued, a TV monitoring study identified a clear predominance of HFSS foods on Austrian TV^(17)^, establishing a baseline for future monitoring studies. The advertised food products were assigned to eight categories (e.g. ‘Fatty, sweet and salty snacks’ or ‘Non-alcoholic beverages’) based on the Austrian Food Based Dietary Guidelines (Food Guide Pyramid); researchers determined whether the ad addressed a general audience or specifically targeted children and the nutritional value of foods and beverages promoted in child-directed ads was then assessed according to EU Pledge Nutrition Criteria^(16)^. The study identified 1919 food advertisements in 360 h of footage: 49·1 % for fatty, sweet and salty snacks (92·4 % in ads targeting children) and 18·8 % (4·1 %) for convenience food. In addition, 95·9 % of ads contained at least one characteristic not in line with nutrition criteria proposed by the industry-led EU Pledge scheme. Most child-directed ads were shown in the evenings on weekdays and in midday hours on weekends.

Since the AVMSD’s 2018 update, the Austrian Advertising Industry Code of Ethics’ 2021 update and the 2021 release of the AT NPM, no studies, to our knowledge, have been published evaluating the TV food marketing landscape in Austria. This study therefore aimed to identify the effects of self-regulatory measures by assessing food and beverage ads shown in 2022 Austrian TV. It applied the AT NPM and WHO EURO NPM version 2015 for nutritional analysis and was performed within the piloting programme of the Best ReMaP EU-wide Joint Action evaluating the feasibility of using the WHO TV monitoring protocol^(18)^ as a monitoring tool in EU Member States^(19)^.

## Methods

This mixed-methods cross-sectional study involved quantitative and qualitative content analyses of advertising on the five most popular TV channels for children and adolescents in Austria, recorded on 4 days per channel. Commercial ads were coded and analysed following the TV monitoring protocol and template V2 2020 (WHO Office for Europe)^(18)^. The nutritional profile of advertised foods and beverages was assessed using both WHO EURO and AT NPM. The AT NPM is slightly stricter and tailored to national needs: some WHO food categories are split into subcategories (e.g. the category ‘Processed meat, poultry, fish and similar’ is divided into four subgroups: ‘Sausages, ham, bacon and similar’; ‘Processed fish, crawfish and molluscs’; ‘Other processed meat, poultry and similar’ and ‘Fried, breaded and pre-baked foods’); others have stricter nutrient thresholds (e.g. ‘Ready-made and convenience foods’ has a sugar threshold of ≤ 7·5 g/100 g in AT, ≤ 10 g/100 g in WHO EURO); some categories are completely new (e.g. ‘Savoury bread spreads’). The AT NPM has been specifically designed to reflect the local food landscape, whereas the WHO EURO NPM is a standardised tool that allows for international comparison. Using both NPM provided an opportunity to test the AT NPM in relation to the WHO EURO NPM.

### Sampling

Data on children’s and adolescents’ most popular TV channels and viewing times were gathered from two Austrian studies on media use^(9,10)^ and on TV usage showing net ranges per group of TV users^(20)^. Peak viewing times, which are defined as times when ‘the number of children watching TV is greater than a quarter of the maximum child audience rating for the day’^(18)^, were as follows: children: 06.30–10.00, 12.00–15.30, 16.00–22.00; teens: 14.00–24.00^(20)^.

The five channels comprised two Austrian (ORF1, ATV2), two German with Austrian commercial breaks for Austrian viewers (PRO7, SuperRTL) and one German with German commercial breaks (Disney Channel). SuperRTL and the Disney Channel are mainly child-oriented, while the other channels serve general audiences. All are free and available throughout Austria; ORF1 is the only public TV station. Private streaming platforms or subscription-based TV channels, such as Netflix or Amazon Prime, were excluded due to the lack of advertising on these platforms at the time of the study.

### Recording

Broadcast footage was recorded for 18 h (06.00–24.00) on two weekdays and two weekend days per channel between 7 December and 18 December 2022, creating a total of 360 h of footage. National holidays, large sporting competitions (multi-day tournaments or seasonal sports leagues), special events or low-rating periods were avoided to provide typical advertising patterns, as advised in the WHO TV monitoring protocol V2 2020^(18)^.

### Coding

All commercials shown in the footage were coded using the extended version of the WHO TV monitoring coding template V2 2020^(21)^, adapted to the Austrian setting by adding teenagers’ peak viewing times and AT NPM thresholds to define marketing eligibility.

If more than one food product, or no product and only the food brand, was shown (‘brand ads’), the WHO TV Monitoring Protocol’s processes were followed: For multiple products, the most dominant one (e.g. in the foreground) was coded. If all products were equally dominant, the first product shown was chosen. In the case of brand ads, the company website was visited, and the most prominent product featured on the homepage was assigned to the ad.

The power of advertisements (creative strategies used to appeal to consumers)^(22)^ was determined by coding persuasive appeals and other strategies, such as jingles, animated characters or links to social media. For every ad, a primary and, if applicable, secondary appeal was identified from the list of twenty-four options in the WHO Protocols. For the complete list of codes, see online supplementary material, Supplemental Table S1.

### Nutritional and data analysis

Nutritional information was obtained from national brand websites, supermarket websites or ‘Food in the Spotlight’^(23)^ – an online tool allowing the comparison of food products available on the Austrian market. Fresh, non-packaged foods were analysed by the Austrian online nutritional software ‘nut.s science’^(24)^.

All products were given a food category code according to WHO EURO NPM; AT NPM provided additional comparative data to determine the prevalence of food ads by food category. Based on these NPM, the products were categorised as not permitted or permitted for marketing to children. Two additional categories not included in either NPM but quite frequently occurring in commercial ads were added to the codebook, namely coffee and alcoholic beverages. Both were classified as not permitted for marketing to children.

Analysis followed the WHO analysis plan^(18)^. After a pre-analysis, in discussion with Joint Action monitoring pilot leads, some amendments were introduced. The primary and secondary appeals’ twenty-four response options were converted to binary variables, each recoded as present/absent. The variable ‘other marketing techniques’ was similarly amended.

The χ2-test was used to evaluate whether proportions of permitted v. non-permitted ads differed, for broadcast time (before/after 18.00) and for presence/absence of persuasive appeals and other marketing techniques. *P* values were set at < 0·05; for multiple comparisons, using the Bonferroni correction, the adjusted *α*-level for persuasive appeals and other marketing techniques was 0·003 and for the marketing technique ‘other characters’ 0·006. All statistical analyses were performed with IBM SPSS Statistics for Windows, version 26.

## Results

### Total number and types of ads

**Figure 1.**
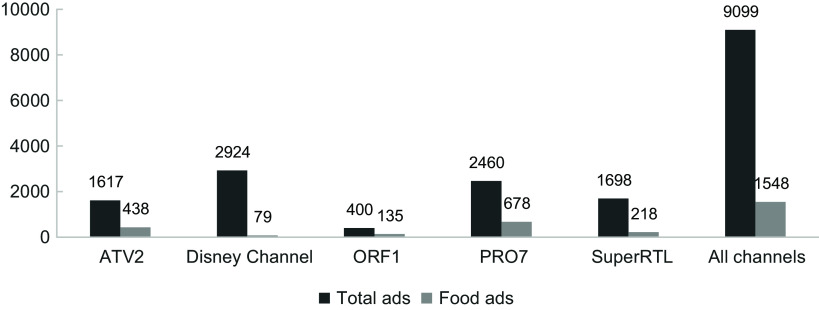
Number of total ads and food ads, by channel and overall.

In total, 9099 advertisements were identified in 360 h of TV. The three German commercial channels had the highest numbers of total ads: Disney Channel (*n* 2924), PRO7 (*n* 2460) and SuperRTL (*n* 1698). Most food ads were shown on German and Austrian commercial channels PRO7 (*n* 678, 27·6 % of PRO7 ads) and ATV2 (*n* 438, 27·1 %). Channels with younger children as the main target audience showed higher total ads, yet lower proportions of food ads (Disney Channel: 2·7 %; SuperRTL: 12·8 %). ORF1, the only public broadcaster, showed fewest (*n* 400) total ads, but the highest proportion of food ads (*n* 135, 33·8 %); see Figure 1.

Across these five channels, 1548 ads (17·0 %) promoted food and beverages, the second most frequently advertised category after toys (24·4 %); on three channels (ATV2, ORF1 and PRO7), food was the most-advertised category. On SuperRTL and the Disney Channel, toys and entertainment were most frequently advertised. Among all food ads, 54·1 % were food company ads featuring one product, 34·2 % food retailer (‘supermarket’) ads, 8·9 % food brand ads and 2·7 % delivery service ads.

### Food ads per food category

**Figure 2.**
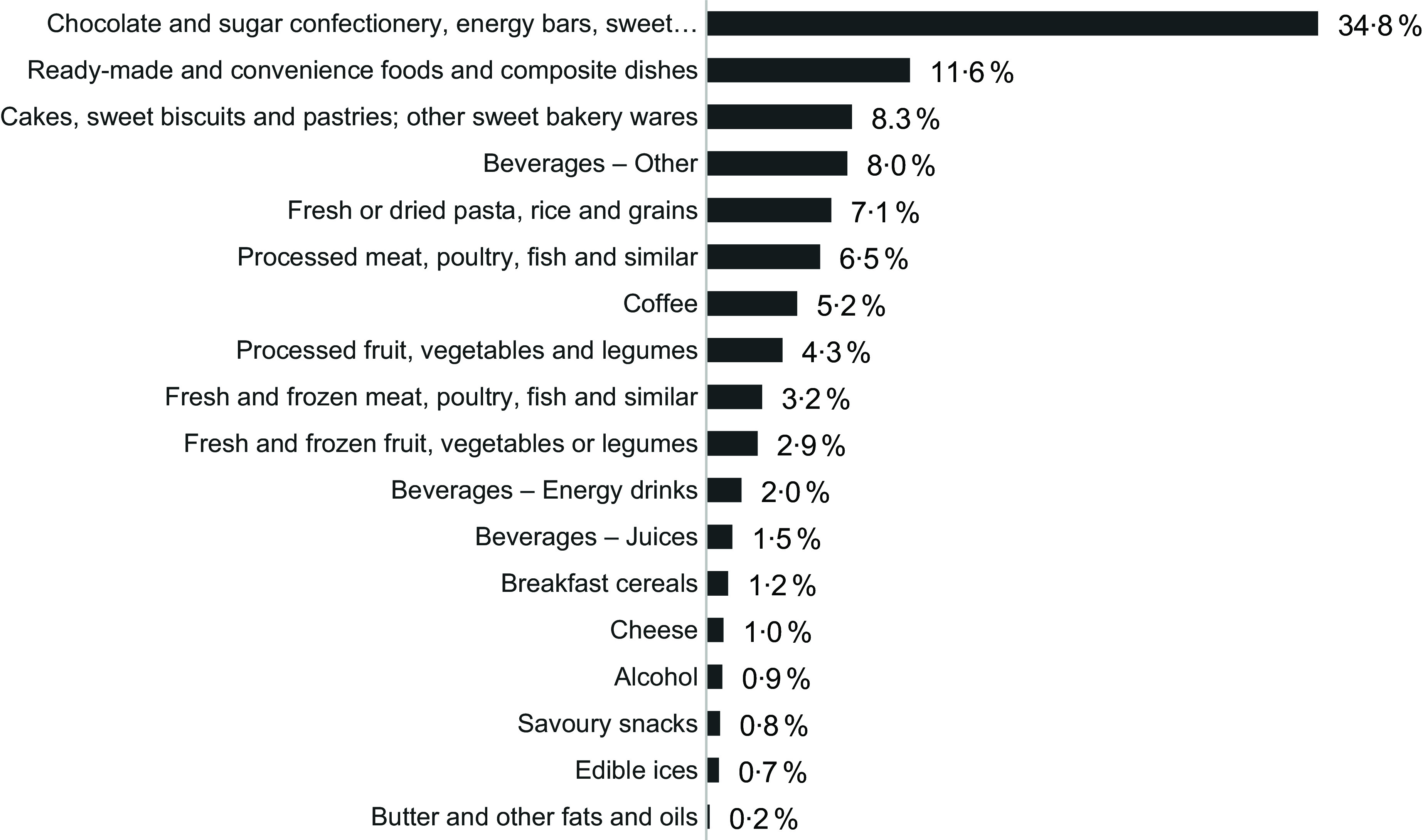
Proportion (%) of food ads per food category according to WHO EURO NPM 2015 (*n* 1548 food ads).

The most frequently advertised WHO EURO NPM food categories across all five channels were: ‘chocolate and sugar confectionary, energy bars, sweet toppings and desserts’ (*n* 538, 34·8 %), ‘ready-made and convenience foods and composite dishes’ (*n* 179, 11·6 %), ‘cakes, sweet biscuits and pastries’ (*n* 128, 8·3 %) and ‘beverages – other’ (*n* 124, 8·0 %), which included cola, lemonade and other sweetened drinks (Figure 2). Fresh, unprocessed foods such as fruit and vegetables, meat or fish each accounted for under 5 % of all ads. Energy drink (*n* 31, 2·0 %), coffee (*n* 80, 5·2 %) and alcohol ads (*n* 14, 0·9 %) were also identified.

Analysis with AT NPM provided similar results, except for a few product groups (e.g. ‘Processed meat, poultry, fish and similar’). For the complete list of food ads per food category according to AT NPM, see online supplementary material, Supplemental Figure S1.

### Eligibility of foods to be marketed to children

In all food ads (*n* 1548), 81·8 % of featured products (*n* 1266) were not permitted for marketing to children according to the WHO EURO NPM.

Food ads with not permitted foods or beverages were significantly more prevalent in the evening, 18.00–24.00 (peak viewing hours for children/teens) than before 18.00 (84·2 % *v*. 79·4 %, *P* = 0·017). The Austrian NPM’s slightly stricter thresholds resulted in a higher prevalence of ads with not permitted foods (83·8 % in total; 85·8 % after 18.00; 81·7 % before 18·00, *P* = 0·033; see online supplementary material, Supplemental Table S2).

The proportion of ads with not permitted foods (WHO EURO NPM) varied between food sectors: 71·7 % (*n* 380) in food retailer (‘supermarket’) ads; 83·3 % (*n* 115) in food brand ads; 87·0 % (*n* 729) in food company ads and 100 % (*n* 42) in food delivery service ads (see online supplementary material, Supplemental Table S2).

### Advertising rate and food advertising rate

**Figure 3.**
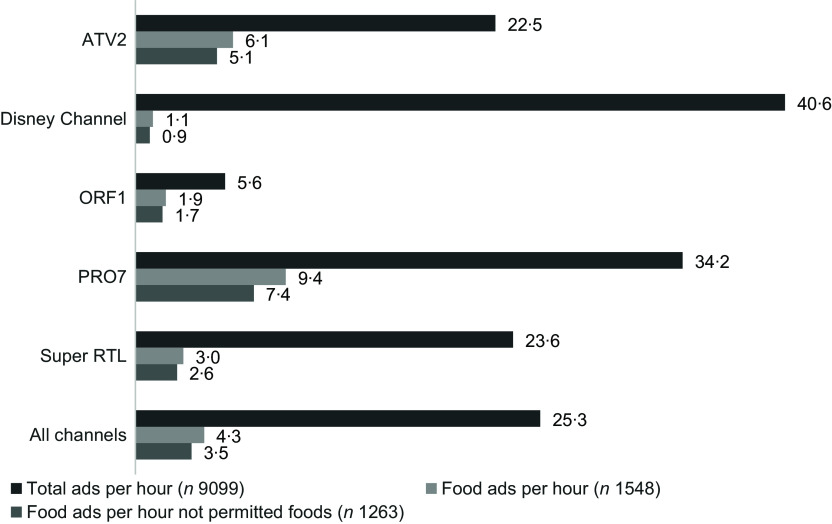
Mean number of total ads/food ads/food ads promoting products not permitted for marketing to children according to WHO EURO NPM 2015, per hour by channel.

The hourly advertising rate (mean ads per hour) across all five channels was 25·3 for all ads (range 5·6 [ORF1] – 40·6 [Disney Channel]) and 4·3 for food ads (range 1·1 [Disney Channel] – 9·4 [PRO7]). The hourly rate of not permitted food ads ranged from 0·9 (Disney Channel) to 7·4 (PRO7) (Figure 3).

**Figure 4.**
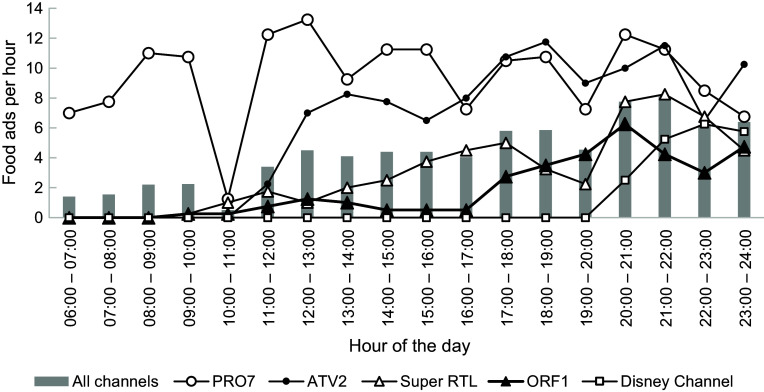
Mean number of food ads per hour of the day, overall and by channel (*n* 1548 food ads).

Figure 4 illustrates the fluctuation of food advertising rates per channel and hours of the day. The total ad and food ad rates increased during the day. Most channels showed no food ads during designated young children’s programming (e.g. 06.00–10.00) but broadcast many after 10.00 and during child/teen peak viewing times.

Both the Austrian public broadcaster (ORF1) and the child-oriented channel with German commercial breaks (Disney Channel) showed few to no food ads during the day until 17.00/20.00 h when the rates picked up. The private channels with Austrian commercial breaks (PRO7, ATV2) showed the highest food advertising rates throughout the day.

### Persuasive appeals

**Figure 5.**
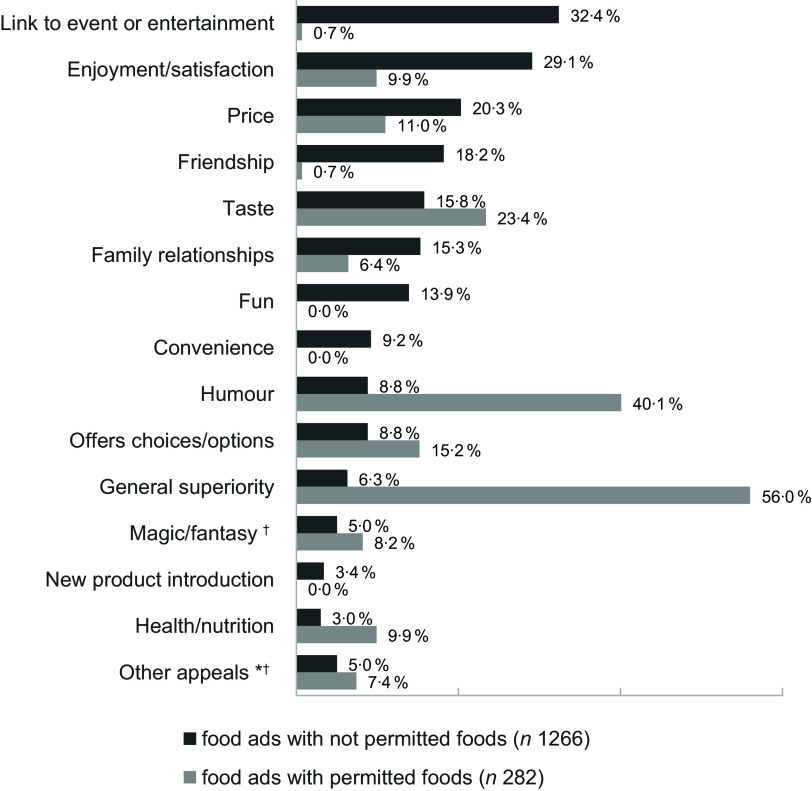
Primary/secondary persuasive appeals used in food ads promoting products not permitted and permitted for marketing to children according to WHO EURO NPM 2015 (in % of food ads for permitted/not permitted foods). *other appeals: corporate information; premium/contest, romance/sex appeal; energy; unique; quantity; holiday, travel or adventure; novel or surprising feature; weight loss/diet; peer status. † Proportions of food ads featuring permitted v. not permitted products did not differ statistically significantly (Bonferroni’s adjusted *α*-level set at 0·003).

Of all the food ads coded (*n* 1548), 93·5 % had more than one persuasive appeal. The observed differences in persuasive appeal between food ads with permitted and not permitted products were significant in all appeals except for magic/fantasy (5·0 % *v*. 8·2 %, *P* = 0·035) and other appeals (5·0 % *v*. 7·4 %, *P* = 0·098). Appeals featuring significantly more often in ads with not permitted than permitted products were, e.g.: link to event or entertainment (32·4 % *v*. 0·7 %, *P* < 0·001), enjoyment/satisfaction (29·1 % *v*. 9·9 %, *P* < 0·001), price (20·3 % *v*. 11·0 %, *P* < 0·001) or friendship (18·2 % *v*. 0·7 %, *P* < 0·001). In contrast, ads with permitted products more often used appeals such as general superiority (6·3 % *v*. 56·0 %, *P* < 0·001), humour (8·8 % *v*. 40·1 %, *P* < 0·001), taste (15·8 % *v*. 23·2 %, *P* = 0·002) or health/nutrition (3·0 % *v*. 9·9 %, *P* = 0·001) (Bonferroni’s adjusted *α*-level was set at 0·003) (Figure 5).

### Food ads appealing to children and teenagers

The proportion of food ads containing products classified as not permitted for marketing to children was higher in those containing elements appealing to children (≤ 12 years) and teens (13–17 years) than without (91·4 % *v*. 76·7 %, *P* < 0·001 and 97·4 % *v*. 76·7 %, *P* < 0·001, respectively).

### Prevalence of other marketing techniques

General marketing techniques, e.g. brand logos, packaging images, musical jingles, occurred quite frequently; digital features, e.g. references to websites or social media, were less prevalent.

**Figure 6.**
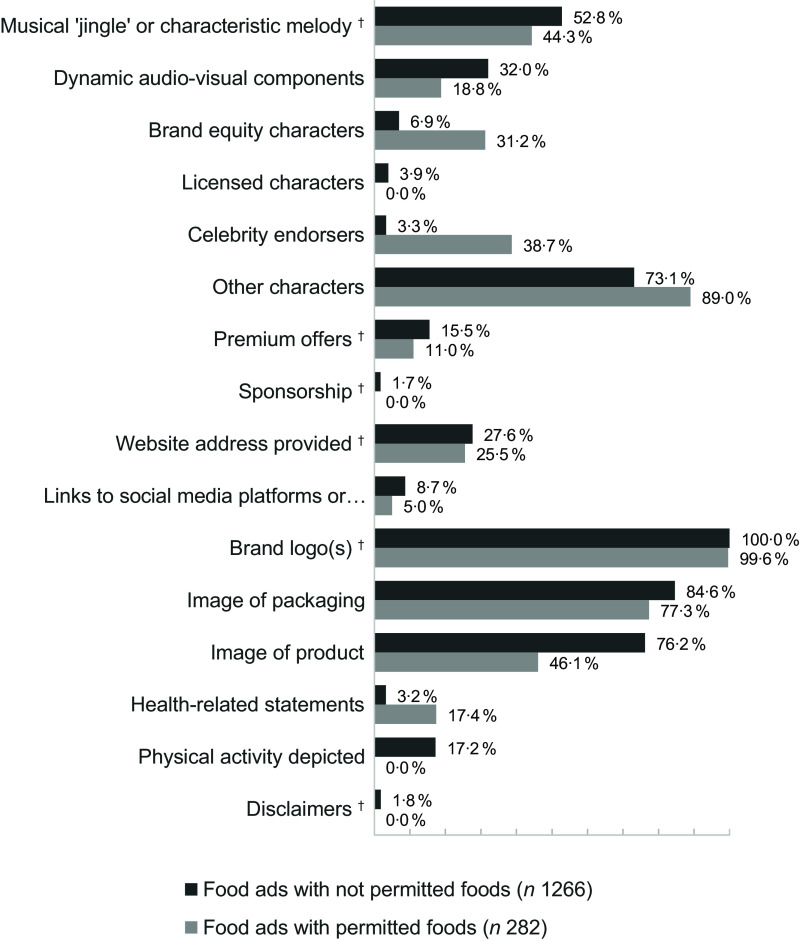
Other marketing techniques used in food ads promoting products not permitted and permitted for marketing to children according to the WHO EURO NPM 2015 (in % of food ads for permitted/not permitted foods). More than one marketing technique per ad possible. † Proportions of food ads featuring permitted v. not permitted products did not differ statistically significantly (Bonferroni’s adjusted *α*-level set at 0·003).

Food ads featuring not permitted products significantly more often used dynamic audio-visual components, i.e. animated graphics or visual effects such as flowing rivers of chocolate or flying superheroes (32·0 % *v*. 18·8 %, *P* < 0·001), images of packaging (84·6 % *v*. 77·3 %, *P* = 0·003) or products (76·2 % v. 46·1 %, *P* < 0·001) and physical activity (17·2 % *v*. 0·0 %, *P* < 0·001) than food ads with permitted products. The use of brand equity characters (6·9 % *v*. 31·2 %, *P* < 0·001), celebrity endorsers (3·3 % *v*. 38·7 %, *P* < 0·001) and health related statements (3·2 % *v*. 17·4 %, *P* < 0·001) was more frequent in food ads with permitted products. In categories such as musical jingle (52·8 % *v*. 44·3 %, *P* = 0·010) or premium offers (15·5 % *v*. 11·0 %, *P* = 0·054) the differences between food ads with permitted and not permitted products were not statistically significant (Bonferroni’s adjusted *α*-level was set at 0·003) (Figure 6).

A detailed analysis of ‘other characters’ revealed that animated characters (5·9 % *v*. 1·1 %, *P* = 0·001), teens (13–17 years) (3·5 % *v*. 0·0 %, *P* = 0·001), young adults (18–25 years) (19·4 % *v*. 8·5 %, *P* < 0·001) and grandparents (4·6 % *v*. 0·0 %, *P* = 0·001) were displayed more in food ads for not permitted products, while food ads for permitted products featured more adults (23·8 % *v*. 58·9 %, *P* < 0·001). Young children (12·8 % *v*. 8·2 %) occurred more in food ads for permitted products, but the association was NS (*P* = 0·016), and no significant association was observed with parents (*P* = 0·285) or older adults (*P* = 0·009) either (Bonferroni’s adjusted *α*-level was set at 0·006).

## Discussion

This study was designed to assess Austrian children’s/teenagers’ potential exposure to TV food and beverage advertisements, and the extent of HFSS advertising after the implementation of self-regulation measures, in a media environment where two-thirds of children and over half of teenagers still watch TV daily^(9,10)^. It was one of the first of its kind in Austria and the first study to use AT NPM^(12)^, WHO EURO NPM^(13)^ and the WHO TV monitoring protocol^(18)^ as reference tools in an Austrian TV monitoring study.

We found that food was frequently advertised on TV channels broadcast in Austria that are popular among children and adolescents, with 17·0 % of all ads promoting food and beverages. This closely matches percentages of food advertising found in similar studies from Russia (19·2 %)^(25)^, Italy (19·4 %)^(26)^ and Germany (17·2 %)^(27)^. While some channels (public broadcaster, child-oriented channel) displayed fewer food ads, all increased the food ad rate towards child/teen peak viewing times in the evening.

Almost all advertised food products (> 80 %) were HFSS and thus not permitted for marketing to children per WHO EURO NPM or AT NPM, consistent with studies from Germany (87·8 %)^(27)^ and Italy (80·7 %)^(26)^. The most frequently-advertised food categories were chocolate, convenience foods, cakes, and sweetened beverages – none of which are classified as permitted for marketing to children according to AT or WHO EURO NPM. Ads for energy drinks, coffee and alcohol (0·9–5·2 % of all food ads) were also identified, despite health risks for children and teenagers^(28,29)^.

Of all food ads, almost 9 % were brand ads (marketing with branding such as a logo, characters or symbols but no identifiable food product), which pose a challenge for food marketing regulation as marketing eligibility is often defined using nutrient thresholds. This is problematic as recent evidence showed that 89 % of over 35 000 products from top 20 food and beverage companies’ sales were classified as ‘unhealthy’^(30)^, and brand marketing can influence preference, choice and purchase intent^(31)^. Applying the product assignment method (as per WHO protocol), this study classified 83·3 % of brand ads as not permitted for marketing to children. Unless this issue is addressed in the design of regulatory measures, children will continue being exposed to brand ads which increase brand recognition and create positive brand attitudes^(31)^.

This study has revealed a distinct profile of advertising strategies used in ads for products that should not be advertised to children according to NPM. Not permitted products were more frequently marketed using emotional marketing strategies such as reference to holidays, friendship, or enjoyment. Permitted products were more often promoted using themes like general superiority, taste or offers choices and rarely with emotional appeals such as fun, friendship or links to events or entertainment (all < 1 %). These findings align with those of Velasquez *et al.* ^(32)^ for TV advertising strategies in Colombia, and are particularly relevant as emotional marketing strategies have been demonstrated in large-scale marketing studies to be the most effective^(33,34)^. Friendship or family was a core theme in many food and beverage ads, exploiting the central role of relationships in people’s lives and thus establishing brand loyalty^(35)^.

Despite avoiding national holidays, intense Christmas-themed marketing occurred throughout the December recording time. However, in the current media and marketing climate, holidays and special occasions are frequently used for marketing over extended periods, thus seasonal advertising has become more a rule than an exception and is therefore worth capturing. Furthermore, marketing around Christmas is often a time of children’s increased exposure and involves particularly powerful child-appealing and emotional advertising strategies. This has since been revised in the WHO protocols, which no longer advise avoiding special days and periods.

The results also showed that TV ads appealing to children and adolescents more often featured HFSS food products than ads not appealing to them. Whereas ads for permitted products more often showed adults and young children, ads promoting not permitted products frequently featured teens and young adults. This might suggest that advertisers have responded to self-regulatory measures, shifting their focus to adolescents^(36)^. Despite calls to protect children up to 18 years^(14)^, adolescents are often not in policy scope assuming that their increased cognitive capacity is protective. There is no evidence, however, for such claims and teenagers remain a vulnerable target, possibly even more so due to greater independence in purchasing and food choices^(37)^. Although small children were less frequently featured in HFSS food ads, there is evidence that the presence of older peers may affect them by modelling their behaviour^(38)^.

Finally, by comparing the results of this study with the 2015 Austrian TV monitoring study^(17)^, the effectiveness of several recently amended restrictions (e.g. EU-Directive 2018/1808, Audiovisual Services Act AMD-G and Code of Ethics of Austrian Advertising Industry) aiming to protect Austrian children from the influence of HFSS food advertising was assessed.

Although methodological differences (e.g. the use of WHO monitoring protocol and NPM *v*. Austrian nutrition guidelines and EU Pledge criteria) mean that like-for-like comparisons cannot always be made, the results indicate that the Austrian TV landscape has changed little in almost a decade despite self-regulatory measures. There were observable changes in the overall number of food ads in the observation period of 360 h (2015: 1919 food ads, i.e. 5·3 ads/hour; 2024: 1548 food ads, i.e. 4·3 ads/hour), yet the vast majority still promoted HFSS food products (2015: 67·9 % in ads for a general audience, 96·5 % in ads targeting children; 2024: 81·8 % in all food ads), and the proportion of ads for healthy foods such as fresh fruit/vegetables continues to be extremely low (2015: 4·5 %; 2024: 5·7 %). The food advertising rate for ads directed at children in 2015 peaked in midday hours on weekends and evenings on weekdays, while in our study the food advertising rate peaked in the evening hours. Both studies showed significant associations between the display of HFSS foods and child appeal and found similar persuasive elements, such as animation, celebrities, fun or music.

These results show that existing regulatory approaches are largely ineffective, and children in Austria are still exposed to high volumes of powerful ads for HFSS food products when watching TV. This is likely because of the significant gaps in current codes. Despite growing evidence that mandatory policy approaches are the most effective in protecting children from harmful food marketing^(15)^, and the most recent 2023 WHO Guideline^(14)^ indicating that effective restrictions should be mandatory, government-led, indicate a NPM to define restricted foods, protect all children under 18 years, and be comprehensive so that migration to other channels and/or age groups is limited, these elements are not currently in place in Austria. All guidelines are still voluntary and focus on designated children’s *programmes*, not *viewing times*, despite viewer data proving that most children and teenagers watch TV in the late afternoon and evening hours – not between 06.00 and 10.00. This was also observed by Lavriša *et al.* ^(39)^, who concluded that regulated times should include peak child viewing. In addition, the code of ethics applies to children under 12 even though teenagers are equally susceptible to marketing strategies^(40)^ and have longer peak viewing times, more similar to adults’^(20)^. Furthermore, none of the guidelines provide clear nutrient benchmarks to determine the healthfulness of foods and beverages advertised.

Other studies have identified the limitations of voluntary industry self-regulation of TV food marketing^(41)^, concluding that nutrition standards are largely ignored, and nutritionally poor food is primarily advertised. A systematic review of forty-four studies on food marketing restrictions concluded that mandatory policies were more likely to be rated as effective than other measures^(15)^. Additionally, a study on the impact of junk food marketing reviewing Euromonitor data showed that mandatory advertising regulations led to a drop in sales per capita (–8·9 %), while self-regulatory measures caused an increase (+1·7 %, *P* = 0·004)^(42)^.

A good example for mandatory marketing restrictions is Chile, where the placement of ‘high-in’ television ads for HFSS foods (similar to WHO’s not permitted ads) was restricted in 2017 by banning these ads in media made for children (or programmes with an audience of at least 20 % below the age of 14) and prohibiting child-directed content such as cartoons, characters or toys in these ads, followed in 2019 by a complete HFSS advertising ban between 06.00 and 22.00^(43)^. This led to a drop of 64 % HFSS food ads on all TV programmes and 77 % during children’s programmes from 2016 to 2019 – the full ban yielding significantly greater results than the 2017 partial ban^(43)^. Subsequently, significant changes in Chilean household food purchase patterns were observed^(44)^.

In South Korea, TV advertising restriction led to an increase in reformulation of HFSS foods; however, there was also a noticeable shift of advertising to online channels^(45)^. In Slovenia, a restriction of child-directed ads on children’s channels led to a shift from children’s channels to general audience channels, which also show programmes watched by children^(39)^.

The German Ministry of Agriculture, Food and Regional Identity proposed a new bill in 2023 demanding that HFSS food ads appealing to children should be prohibited in all relevant media between 06.00 and 23.00 and that WHO EURO NPM should be used for nutrient thresholds^(46)^. Unfortunately, this bill has not been passed yet, and its future is uncertain. To date, Germany relies on self-regulation like Austria, except protecting children below 14 years instead of 12^(47)^. This may be why German TV stations such as the Disney Channel show fewer food ads during daytime. However, the current regulatory system yields very similar results to Austria^(27)^.

Policymakers in Austria and globally should consider increased regulation of HFSS food advertising during child/teen viewing times to prevent the growing obesity epidemic and avoid early HFSS food brand preference development. Since this does not only concern one member state alone, and emerging digital marketing requires cross-border solutions, a common strategy for all EU member states would be a necessary approach.

Further studies ought to determine current child and teen media use to obtain more baseline data and focus on the ever-growing field of digital and social media, alongside traditional broadcasting and TV streaming.

## Conclusion

This study has shown that Austrian TV advertising on channels popular among children contains high levels of food and beverage ads at almost all times analysed, including child/teen peak viewing times. Most advertise HFSS food products not permitted for marketing to children according to WHO EURO NPM and AT NPM. Many highly persuasive, child and teen-appealing elements and marketing strategies are used, especially for HFSS foods. This indicates that self-regulation is not sufficiently effective in reducing children’s exposure to food marketing on TV in Austria, and children remain exposed to HFSS food marketing content that affects their health and violates children’s rights. Mandatory, government-led policy measures (e.g. addressing all children under 18 years, using an NPM to define restricted foods, comprising all types of media and children’s viewing times), accompanied by comprehensive monitoring, are needed to effectively protect children and adolescents from the harmful effects of HFSS food marketing.

## Supporting information

Moll et al. supplementary material Moll et al. supplementary material

## Supplementary material

For supplementary material/s referred to in this article, please visit https://doi.org/10.1017/S1368980026102171
