# Supplementary material for: Children still exposed to high rates of unhealthy food advertising in Austria: does self-regulation work?
Source: Public Health Nutr. 2026 Feb 26;29(1):e57. doi: 10.1017/S1368980026102171 (PMC13112307; doi:10.1017/S1368980026102171)
Supplement: Moll et al. supplementary material [file S1368980026102171sup001.docx]

**Supplementary Material**

Supplemental Table S1: Complete list of codes (based on WHO TV Monitoring Protocol, unpublished)

| **Variable** | **Coding** |
| --- | --- |
| **Variable Type: EXPOSURE** |  |
| **ALL ADS** | |
| - Country |  |
| - Channel name | 1 = ATV2, 2 = Disney Channel, 3 = ORF1, 4 = PRO7, 5 = SuperRTL |
| - Date the marketing activity occurred |  |
| - Day of the week the marketing occurred |  |
| - Programme category | 1= Comedy; 2 = Drama; 3 = Movie; 4 = Soap opera; 5 = Music/music video; 6 = News/commentary; 7 = Talk show; 8 = Reality; 9 = Sports; 10 = Entertainment/variety; 11 = Documentary; 12 = Game; 13 = Children's; 14 = Infomercial; 15 = Retail; 16 = Other |
| - Programme Name |  |
| - Programme Start Time |  |
| - Advert time slot (per 1/2 hour) | 1 = 06:00 to 36 = 23:30 |
| - Peak or non-peak children's viewing time | 1 = peak viewing time (6:30-10:00, 12:00-15:30, 16:00-22:00); 0 = non-peak |
| - Peak or non-peak teens' viewing time | 1 = peak viewing time (14:00-24:00); 0 = non-peak |
| - Between or within programme | 1 = within programme; 0 = between programme |
| - Advertised product type | 1 = Food and drink; 2 = Clothes/shoes; 3 = Education; 4 = Entertainment (including music, video, films, entertainment parks); 5 = Financial (including building societies, banks, insurance, pensions); 6 = Household cleaners/detergents (including washing up liquid, washing powders, cleaning fluids); 7 = Household equipment (including electrical appliances); 8 = Motoring (including cars and petrol); 9 = Pet products (including pet food); 10 = Pharmaceuticals (including medications, vitamin pills, breath fresheners); 11 = Public information announcements/community service announcements (general); 12 = Public information announcements (sponsored by food companies); 13 = Publishing (including magazines, books, newspapers, recipe books, cooking magazines); 14 = Retailing and mail order (including catalogues, *not* including supermarkets – supermarkets coded as a food ad); 15 = Toiletries (including soap, hair shampoo, cosmetics, nappies, sanitary items); 16 = Toys; 17 = Travel/transport/holidays; 18 = Utilities (including telephone, gas, electricity, mobile/cell phones); 19 = Channel promotions (including promotions for other programmes on the same channel, or on other channels); 20 = Other (e.g. lottery, sports betting, matchmaking services) |
| **FOOD or BEVERAGE ADS** | |
| - Food product brand name |  |
| - Food brand ad, food retailer ad, food company ad or delivery service ad | 1 = food brand ad; 2 = food retailer („supermarket“) ad; 3 = food company ad (featuring at least one product); 4 = delivery service ad |
| - Detailed description of food product |  |
| - WHO EURO NPM 2015 Food category code | 1 = Chocolate and sugar confectionery, energy bars, sweet toppings and desserts; 2 = Cakes, sweet biscuits and pastries; other sweet bakery wares, and dry mixes for making such; 3 = Savoury snacks; 4a = Beverages – Juices; 4b = Beverages - Milk drinks; 4c = Beverages - Energy drinks; 4d = Beverages – Other; 5 = Edible ices; 6 = Breakfast cereals; 7 = Yoghurts, sour milk, cream and other similar foods; 9 = Ready-made and convenience foods and composite dishes; 10 = Butter and other fats and oils; 11 = Bread, bread products and crisp breads; 12 = Fresh or dried pasta, rice and grains; 13 = Fresh and frozen meat, poultry, fish and similar (Includes eggs); 14 = Processed meat, poultry, fish and similar; 15 = Fresh and frozen fruit, vegetables or legumes; 16 = Processed fruit, vegetables and legumes; 17 = Sauces, dips and dressings;  Additional categories: 18 = Coffee; 19 = Alcohol |
| - Austrian NPM 2021 Food category code | 1 = Chocolate and sugar confectionery, energy bars, sweet toppings and desserts; 2 = Cakes, sweet biscuits and pastries; other sweet bakery wares, baking mixtures and doughs; 3 = Edible Ices; 4 = Savoury snacks; 5a = Drinking water, bottled water; 5b = Juices; 5c = Energy drinks; 5d = Other beverages; 6 = Breakfast cereals; 7 = Bread, baked goods, baking mixtures and doughs; 8 = Plant-based drinks, plant-based desserts and similar; 9a = Milk and mixed dairy products from non-fermented milk; 9b = Yoghurts, sour milk, cream, cream mix and other similar foods; 9c = Mature cheese (hard, semi-hard, soft and sour milk cheese), firm Mozzarella; 9d = Fresh cheese and other cheese; 10 = Savoury bread spreads; 11 = Nuts and seeds; 12 = Fats and oils; 13 = Ready-made and convenience foods and composite dishes, baking mixtures and doughs; 14 = Pasta, rice and grains; 15 = Fresh and frozen meat, poultry, fish and eggs; 16a = Sausages, ham, bacon and similar; 16b = Processed fish, crawfish and molluscs (neither breaded nor fried); 16c = Other processed meat, poultry and similar (neither breaded nor fried); 16d = Fried, breaded and pre-baked foods; 17 = Fresh and frozen fruit, vegetables and legumes; 18 = Processed fruit, vegetables and legumes (neither breaded nor fried); 19 = Sauces, dips and dressings  Additional categories: 20 = Coffee; 21 = Alcohol |
| - Total fat | grams per 100g/ml product |
| - Saturated fat | grams per 100g/ml product |
| - Total sugars | grams per 100g/ml product |
| - Added sugars | 1 = present; 0 = absent |
| - Non-sugar sweeteners | 1 = present; 0 = absent |
| - Salt | grams per 100g/ml product |
| - Energy | kcal per 100g/ml product |
| - Fibre | grams per 100g/ml product |
| - Marketing permitted according to WHO EURO NPM 2015? | 1 = yes; 0 = no |
| - Marketing permitted according to Austrian NPM 2021? | 1 = yes; 0 = no |
| - Notes (URLs of sites used in determining nutritional information) |  |
| **Variable Type: POWER** |  |
| **FOOD or BEVERAGE ADS** | |
| - Primary persuasive appeal (the main theme or topic of the advertisement) | 1 = quantity (e.g. "now in an even bigger pack"); 2 = convenience (e.g. "easy to eat on the go"); 3 = taste (e.g. "great tasting snack"); 4 = health/nutrition (e.g. "packed full of vitamins"); 5 = energy (e.g. "this product will give you a burst of energy"); 6 = price (e.g. "only 99p"); 7 = unique (e.g. "there's no other snack like it!"); 8 = fun (e.g. the product is fun, eating it is fun); 9 = family relationships (e.g. the product being at the centre of bonding or love between family members); 10 = general superiority (e.g. "the best chocolate bar there is"); 11 = peer status (e.g. "be the envy of your friends!"); 12 = friendship (e.g. "spend time with friends", or emphasises a friendship); 13 = romance/sex appeal; 14 = premium/contest (e.g. "if you buy this product, you could be in with a chance of winning"); 15 = weight loss/diet (e.g. "low fat" or "now only 100 calories"); 16 = offers choices/options (e.g. "has 12 different flavours"); 17 = enjoyment/satisfaction (e.g. shows people eating the product and smiling); 18 = new product introduction (e.g. "introducing this new snack, try it!"); 19 = corporate information (e.g. "this snack is brought to you by brand XY"); 20 = humour (e.g. the ad has jokes in it); 21 = magic/fantasy (the ad contains characters from fairy tales, stories, or surprising events such as disappearing or floating objects); 22 = link to event or entertainment (ad features a music, child-focused, sporting or other event); 23 = holiday, travel or adventure; 24 = novel or surprising feature |
| - Secondary persuasive appeal (another prominent persuasive theme) | 0 = only a primary appeal identified, no secondary appeal noted; 1 to 24 (see primary persuasive appeal) |
| - Musical 'jingle' or characteristic melody | 1 = present; 0 = absent |
| - Dynamic audio-visual components | 1 = present; 0 = absent |
| - Brand equity characters (characters created for the product or brand) | 1 = present; 0 = absent |
| - Licensed characters (characters created for a movie/TV-show, then licensed by a brand for promotional purposes) | 1 = present; 0 = absent |
| - Celebrity endorsers | 0 = none; 1 = entertainment celebrity; 2 = sports person; 3 = business leader; 4 = politician; 5 = Internet celebrity (e.g. YouTuber, "influencer"); 6 = other (make a note on the file to specify) |
| - Other character | 0 = no; 1 = non-human animated character; 1 = animated child-like human character; 2 = animated adult-like human character; 3 = young child (<12 years); 4 = teen (13 - 17 years); 5 = young adult (18 - 25 years); 6 = adult (mid-twenties +); 7 = parent (any age); 8 = grandparent (any age); 9 = older adult (e.g. retired, 60s+) |
| - Premium offers (e.g. giveaways, collectibles, competitions, contests, vouchers and rebates) | 1 = with premium offers; 0 = without |
| - Sponsorship (e.g. sponsoring a sports team, school or cultural event) | 1 = sponsorship mentioned verbally or visually, 0 = without |
| - Website address provided | 1 = mentions the company website, flashes the website on screen; 0 = none |
| - Links to social media platforms or campaigns | 1 = has Twitter hashtag, shows Facebook group/presence, suggests joining online 'club' etc; 0 = none |
| - Brand logo(s) | 1 = displayed on screen during ad; 0 = absent |
| - Image of packaging | 1 = displayed on screen during ad; 0 = absent |
| - Image of product itself (the food or drink) | 1 = displayed on screen during ad; 0 = absent |
| - Health claims (verbal or textual) | 0 = none; 1 = low fat/fat free; 2 = sugar free; 3 = no added sugar/less sugar; 4 = low calorie/light; 5 = low carbohydrate; 6 = organic; 7 = natural ingredients/all natural/no preservatives/nothing artificial; 8 = provides essential nutrients (including protein, calcium, potassium, vitamins, antioxidants); 9 = whole grain/whole wheat; 10 = fibre or bran; 11 = heart healthy/low cholesterol; 12 = diet; 13 = "five a day"; 14 = healthy food (e.g. ice cream or chocolate are made of milk, smoothies contain the goodness of fruit); 16 = other (make a note on the file to specify) |
| - Physical activity depicted (characters shown in purposeful physical activity, e.g. running, jumping, playing sport; beyond casual walking or moving) | 1 = physical activity depicted; 0 = none depicted |
| - Disclaimers | 0 = none; 1 = part of a balanced/complete/nutritious breakfast or meal; 2 = part of a balanced/healthy diet; 3 = not a substitute for a real meal; 4 = enjoy in moderation; 5 = other |
| - Which demographic group does the ad appeal to most? | 1 = children (12 years and under); 2 = children and teens (all <18 years)  3 = teens (13 - 17 years); 4 = teens (13 - 17 years) and young adults (18 - 25 years); 5 = young adults (18 - mid-twenties); 6 = adults (mid-20s +); 7 = parents (any age); 8 = grandparents (any age); 9 = older adults (e.g. retired, 60s+); 10 = all ages; 11 = families |
| - Who else does the ad appeal to? | 0 = just the group already coded; 1 to 11 (see above) |
| - Does the ad appeal to children (12y and under)? | 1 = yes; 0 = no |
| - Does the ad appeal to teens (13-17y)? | 1 = yes; 0 = no |

Supplemental Figure S1: Proportion (%) of food ads per food category according to Austrian NPM 2021 (n 1548 food ads)

Supplemental Table S2: Proportion (%) of products in food ads permitted or not permitted for marketing to children according to WHO EURO NPM 2015 and Austrian NPM 2021, by food sector, channel and time

|  | Products not permitted to be marketed to children (WHO EURO NPM) | | Products permitted to be  marketed to children (WHO EURO NPM) | | Products not permitted to be marketed to children (AT NPM) | | Products permitted to be  marketed to children (AT NPM) | | Total | |
| --- | --- | --- | --- | --- | --- | --- | --- | --- | --- | --- |
|  | n | % | n | % | n | % | n | % | n | % |
| Total food ads | 1266 | 81.8% | 282 | 18.2% | 1297 | 83.8% | 251 | 16.2% | 1548 | 100.0% |
| Food ads by food sector |  |  |  |  |  |  |  |  |  |  |
| Food brand ads | 115 | 83.3% | 23 | 16.7% | 115 | 83.3% | 23 | 16.7% | 138 | 100.0% |
| Food company ads | 729 | 87.0% | 109 | 13.0% | 758 | 90.5% | 80 | 9.5% | 838 | 100.0% |
| Food delivery service ads | 42 | 100.0% | 0 | 0.0% | 42 | 100.0% | 0 | 0.0% | 42 | 100.0% |
| Food retailer ads | 380 | 71.7% | 150 | 28.3% | 382 | 72.1% | 148 | 27.9% | 530 | 100.0% |
| Food ads by channel |  |  |  |  |  |  |  |  |  |  |
| ATV2 | 364 | 83.1% | 74 | 16.9% | 393 | 89.7% | 45 | 10.3% | 438 | 100.0% |
| Disney Channel | 62 | 78.5% | 17 | 21.5% | 62 | 78.5% | 17 | 21.5% | 79 | 100.0% |
| ORF1 | 122 | 90.4% | 13 | 9.6% | 124 | 91.9% | 11 | 8.1% | 135 | 100.0% |
| PRO7 | 531 | 78.3% | 147 | 21.7% | 531 | 78.3% | 147 | 21.7% | 678 | 100.0% |
| SuperRTL | 187 | 85.8% | 31 | 14.2% | 187 | 85.8% | 31 | 14.2% | 218 | 100.0% |
| Food ads by time |  |  |  |  |  |  |  |  |  |  |
| 06.00 – 18.00 | 612 | 79.4% | 159 | 20.62% | 630 | 81.7% | 141 | 18.3% | 771 | 100.0% |
| 18.00 – 24.00 | 654 | 84.2% | 123 | 15.83% | 667 | 85.8% | 110 | 14.2% | 777 | 100.0% |
